# Interoception and Emotion: A Potential Mechanism for Intervention With Manual Treatment

**DOI:** 10.7759/cureus.15923

**Published:** 2021-06-25

**Authors:** Hugo Pasin Neto, Eduardo Bicalho, Gustavo Bortolazzo

**Affiliations:** 1 Osteopathy, Brazilian College of Osteopathy, Sorocaba, BRA; 2 Physiotherapy, University of Sorocaba, Sorocaba, BRA

**Keywords:** interoception, emotional responses, visceral manual treatment, central sensitization, emotional imbalances

## Abstract

Interoception is considered a perception pathway as important as the exteroceptive pathways for determining responses to maintain homeostasis. There is evidence about the influence of the interoception on emotional responses as these expressions are considered to be a combination of physical, environmental and individual beliefs. A large percentage of afferent fibers in the body are related to free nerve endings which, when stimulated, reach the insular cortex that participates in the process of emotions. The viscera afferent fibers represent 5% to 15% of all these inputs. Evidence emerges that demonstrates the importance of visceral health as part of the treatment of patients with emotional imbalances. It can be postulated that manual treatment applied to visceral fasciae can assist in interoceptive balance and have a positive impact on emotions. Therefore, the objective of the present study is to discuss the concepts of interoception, central sensitization, emotional health and visceral manual treatment.

## Introduction and background

Defined by Craig [1], as “the physiological sense of the body conditions”, interoception is considered the internal input of the body by several other authors [2-5]. These interoceptive afferents originate throughout the body, especially in the viscera [1]. Hobday et al. demonstrated that the stimulus in the esophagus, large intestine and rectum (not anus) generates activation of the insula and cingulate cortex, as well as in the prefrontal cortex [6].

Dysfunctional afferents can lead to central sensitization as defined by the International Association for the Study of Pain (IASP) as the increase in the response capacity of nociceptive neurons in the central nervous system (CNS) [7]. The insula, cingulate and prefrontal cortex form a sensory network that involves the reception of interoceptive sensation, the awareness of that feeling, and the elaboration of the motor component to express those feelings [8]. This suggests the possibility that visceral treatment may interfere with patients' emotional conditions by modulating the polymodal receptors present in the visceral fasciae.

This hypothesis could be supported by Dum et al. [9] who stated that interoception participates in emotional processes, as well as Naring who demonstrated this relationship with a variety of anxiety addiction [10], and also D 'Alessandro who related to eating disorders [11]. In addition, Pollatos et al. stated that individuals with good interoceptive sensitivity have more intense emotional experiences [12].

Therefore, the objective of the present paper is to present a theoretical framework for this proposal, serving as a basis for clinical application, as well as for future clinical trials related to the theme.

## Review

Sensitization

This condition is defined by the IASP as an increase in the response capacity of nociceptive neurons in the CNS to their normal or subliminal afferent entry [7]. There are two types of sensitization involved: central and peripheral. Central sensitization involves broad CNS processes. It occurs when harmful stimuli in receptors located in any peripherally innervated sensory tissue, maintained over time, promote neuroplasticity events with the increased excitability of neurons involved in central nociceptive activity and in the upper centers responsible for the perception of pain [13,14]. Peripheral sensitization is a phenomenon that occurs at the local tissue level, and involves modification in the activity of multimodal sensory receptors promoting allodynia conditions, when a non-nociceptive stimulus causes the perception of pain, and/or also hyperalgesia, in which a nociceptive stimulus causes the excessive sensation of pain [15-17]. The sensitization process is reversible and can be modulated. Under normal conditions, when tissue repair occurs, nociceptive bombardment ceases and the phenomenon is resolved spontaneously [18]; however, it can last beyond the trigger factor caused by the injury or inflammation of a peripheral tissue, affecting the CNS (bottom-up), in such a way that it could be maintained by superior activities of the SNC (top-down) [19]. Experimental studies have shown that central sensitization can be promoted by primary afferent inputs originating from sensory receptors in somatic and also visceral tissues [20-22].

The viscera have a complex system of vagal and spinal innervation (sympathetic), with about 5% to 15% of all afferent inputs to the spinal cord originating from the viscera [23]. Visceral afferents are multimodal, that is, they are mechanosensitive, chemosensitive and thermosensitive, as well as having the potential for sensitization in the majority. It is known that many nociceptors, including visceral ones, are also responsive to low threshold non-nociceptive stimuli, such as mechanical stimuli [23]. Pain originating in the organs involves stimuli produced by conditions such as stretching/distention of hollow organs, mesentery traction, ischemia/hypoxia and chemical stimuli promoted by inflammatory mediators. Cervero [24] and Anand et al. [25] highlighted the roles of visceral multimodal receptors possibly involved in the phenomenon of peripheral and central sensitization [24]. Previously it was postulated that the parasympathetic afferent functions would be related only to autonomic control and not to perceptions and sensations, while the sympathetic system would be responsible for the conduction of pain sensations and other visceral sensations. The roles of vagal innervation on the modulation mechanisms in pain transmission and its analgesic effects are currently known; however, their role in visceral nociceptive transmission remains open [23].

In recent years, there has been some speculation about the sensitization conditions addressed clinically and the impact of this phenomenon on interoceptive physiological perception. D´alessandro et al. proposed that there are close relationships between sensitization and interoception phenomena, and that these conditions can help to support important concepts for the better interpretation of different clinical scenarios [11]. The authors suggest a theoretical framework called the “interoceptive paradigm” that takes into account possible physiological interferences that manual approaches, especially Osteopathy, can produce in conditions of central sensitization through interoceptive paths involved in these approaches.

Interoception

In the 19th century, the means by which the body communicated with the brain gave rise to the concept of coesthesia, defined at the time as an unconscious information about the normal functioning of the body and its organs that would become conscious only in the presence of the disease on this system [5]. Recently, this concept was revised and called interoception [1].

There are many definitions related to the term interoception. The pioneer Sherrington, defines as “the sensory input of receptors located inside the body”. More current definitions include “the emotional motor system” [25], or the one of Cameron who considers that interoception is a multidimensional construction, which includes how people evaluate and react to body sensations [2]. Finally, Craig defines it as an ongoing homeostatic and sensory pathway of the autonomic nervous system [3].

Unlike what happens in the search for a better definition, the interoceptive path is a consensus among different authors, being considered an evolution in anthropoid primates. This "direct" path between the spinal cord, thalamus and cortex is responsible for this condition.

It is worth noting that the final destiny of this path would not be the primary sensory region of the cortex, a place where the proprioceptive receptors reach, but rather, specific locations of the insular cortex [26], among other regions. Another important observation is related to the number of receptors related to this interoceptive function, demonstrating the importance of this influence for the maintenance of homeostasis.

Of all the afferent fibers in the body, 80% are related to free nerve endings of which 90% are slow-conducting C fibers which [27], according to Schleip et al. [28], when stimulated reach the insular cortex. Thus, through primary afferent a-delta fibers of small diameter and afferent fibers C, the physiological status of each tissue in the body is represented. These fibers carry information related to the mechanical, thermal, chemical, metabolic and hormonal status of all the body, including the viscera [29]. These fibers end monosynaptically in lamina I, the most superficial layer of the spinal cord. From this region, lamina I neurons project to the lateral column, where the bodies of the pre-ganglionic neurons of the sympathetic autonomic nervous system are present, and to pre-autonomic sites in the brain stem [30], targeting the nucleus of the solitary tract that also receives visceral and gustatory information through the vagus, glossopharyngeal and facial nerves. Strigo, Craig and Smith et al. [31,32] reinforce this relationship, citing that visceral homeostatic sensory activities are also mediated by the entry of the vagus nerve in this region.

In the brain stem, in addition to the nucleus of the solitary tract, previously mentioned as the point of entry for several important nerves for interoception, there are many places of note due to the presence of afferent pathways [33], descending pathways [34] and local modulatory networks, which through neurotransmitters contribute to the elaboration of emotion and emotional behavior [35], even before if reaching higher levels [5], as well as participating in the integration/excitation of upper structures, it can be called ascending reticular activation or ascending excitation network [36,37].

With regard to these nuclei, the parabrachial is the first one. According to Damasio and Carvalho [38], this would be the most caudal structure related to the integration of interoceptive information. Saper [39], reinforce this importance, stressing that this would be the main point of integration for all homeostatic afferents, participating in an important way in maintaining cardiovascular, respiratory, energetic balance (food and glucose) and liquid (electrolyte and water). Screening studies have shown that there are upward projections of the nucleus of the solitary tract and direct projections of lamina I neurons to this region, which in turn sends fibers to the periaqueductal gray substance [39-42], thalamus, hypothalamus and amygdala [37].

The periaqueductal gray substance, mentioned above as one of the projections of parabrachial nuclei, is located in the midbrain, surrounding the cerebral aqueduct and is an important point of integration of affective and autonomic behavior [43]. Several fixed patterns of behavior with altered autonomous responses, particularly those related to responding to external threats, are related to their longitudinal columns (dorsomedial, dorsolateral, ventrolateral and lateral) [44], which can be considered fundamental structures in the processing of coping strategies for different types of stress. This function is due to the fact that this region is a true anatomical and functional interface between the cortex, the brainstem and the ascending pathways, modulating and integrating the information that arrives from the spinal cord and reaches the brain areas that meet the affective-motivational component [45].

The thalamus, in turn, contains multiple structures that appear to play an essential role in emotional processing from the brain stem to the cerebral cortex [5]. Among the nuclei present in the medial regions of the thalamus, we highlight the posteromedial ventral nuclei, which receive projections from the parabrachial complex and send to the insular cortex, the intralaminar nuclei, which send signals to the orbitofrontal and anterior cingulate cortices. The posteriormedial ventral thalamic nuclei also receive pathways that arrive directly from the spinal cord [5].

Starting from the aforementioned brainstem nuclei, several multidirectional connections occur with the cortex, including the hypothalamus, the cingulate cortex, the insula and the amygdala. According to Benarroch [46], this whole system can be called the "central autonomic network" and is composed of several regions of the cortex and brainstem, in addition to those previously mentioned, such as the prefrontal cortex, the orbitofrontal cortex, the ambiguous nucleus, the dorsal motor nucleus of the vagus, the locus coeruleus, among others. This complex network was shown by retrograde staining studies [9] that evaluated the connections between vagal and sympathetic neurons with this series of brain regions, which are hierarchically organized.

Upon reaching this “central autonomic network”, especially the anterior insula and the cingulate cortex, a sensory network is formed in which they involve the reception of sensation, as well as the awareness of that sensation and then the elaboration of the motor component to express a feeling [47].

The insula, considered one of the main members of this network, can be didactically organized from posterior to anterior, with the posterior part corresponding to primary sensory input, such as gustatory, vestibular and visceral sensations [1]. Nguyen et al. evidenced this relationship through direct stimulation of the human insular cortex, with implanted electrodes, and observed somatosensory, viscerosensory, motor, auditory, vestibular and speech responses [48]. Other authors corroborate this idea, such as Khalsa et al. that reported that the insula is related to the awareness of bodily sensations, in a way that allows authors such as Azanon et al. and Ibanez and Manes state that it is a point of convergence between the internal and external environment [49-51].

These anatomical and physiological studies support the ideas of some authors, such as Schleip et al. that describe this network as the key to the integration between the perception of the body and the mental processes, proposing that human beings are able to use non-conscious physical sensations at the moment of decision-making. After all, these afferences would bring the possibility of affective integration in the search for homeostasis [28].

Interoception and emotions

According to Smith et al. [32], higher-level functions may depend on lower-level functions. These authors suggest that before anyone can worry about meeting cognitive/emotional needs, it is necessary that metabolic resources are maintained at adequate levels. This statement makes clear the importance of interoception in the regulation of emotional expression, reinforced by DʻAlessandro who claims that it would be able to influence all levels of the central nervous system through awareness, being potentially important for the relationship between physical health and mental [11].

According to Damasio et al. [52], this relationship is due to the fact that the condition of the body of each individual, called homeostatic processing, would be one of the bases for the construction of the “I” in the elaboration of emotional awareness. This idea corroborates with the proposal by Cameron who stated that the expression of emotion would be linked to the evaluation of sensations and the way in which the individual reacts to them [2].

In this sense, Paulus and Stein reported that disorders such as anxiety and depression can be related to the sum of an interoception and states of dysfunctional beliefs [53]. Exemplifying this relationship, Barrett describes that “at a given moment, within a given context interpreted by your memory and belief, your brain uses concepts to make sense of internal sensations, all simultaneously, that is, from a sore stomach, your brain builds an example of hunger, nausea or distrust” [54]. Still in this line, Wiens and Herbert et al. report that the dysfunctional interoception could have an effect on cognitive and behavioral functions [55,56]. Herbert and Pollatos reported that interoception influences the attention process, decision making and eating disorders, respectively, making it evident that emotions can be related to the integrated activities of neural networks that make up the entire system interoceptive, from lower to higher levels [57].

In studies that evaluate these responses, Menon and Uddin stated that the insular and cingulate cortices are activated together during all the affective bodily feelings, acting as a “control network” and would be dysfunctional in people with mental disorders [58]. According to Craig [1], imaging studies report this joint activation in individuals subjected to emotional feelings, such as maternal love, anger, fear, sadness, happiness, sexual arousal, among others, demonstrating that the anterior insular cortex is activated in all subjective feelings, being defined by the author as “the generator of human consciousness”.

In another study, Holstege analyzed the behavior of the brain and brainstem in humans and showed that there is a relationship between decreased activation of the periaqueductal gray substance and sexual desire disorder in women [4]. According to the author, this was due to a hypoactivity of the orbitofrontal cortex, another region considered essential with regard to the control of emotional expression and human behavior.

Observing all this integration, as presented in the previous paragraphs, it is evident that no matter how many experiments try to isolate certain regions and relate them to a certain behavior, this notion of specific representations of emotions in the brain has been replaced by a much more integrative concept, understanding that it is a more complex network based on external and internal stimuli. This idea has been supported by neuroimaging data that show that the affective dimensions are distributed and integrated in cortical and subcortical regions in such a way that it is possible to perceive that there is a bidirectional interaction between each of the levels described above [59].

Barret corroborates this idea and states that our brain acts as a completely integrated network that contributes to the interpretation and elaboration of different mental states, considering the idea that most neurons are multipurpose, that is, there is no region of the brain related to a single emotion. According to the author, emotions arise from the firing of neurons, but no neuron is dedicated exclusively to emotion. This idea reinforces the importance of a balanced interoception to contribute to the construction of balanced emotional responses [54].

Responses of the manual treatment on interoception

The viscera can present dysfunctions due to structural, thermal and chemical factors, and also due to a lack of flexibility and viscoelasticity [60]. Consequently, these dysfunctions may interfere negatively in interoception, since multimodal receptors change its behaviour conducting mostly nociceptive stimuli, generating a sensitization state of the nervous system [11]. Therefore, approaches that aim to reduce peripheral and central sensitization states can improve interoceptive afferences and may have a positive impact on emotional behavior. Among the possibilities, manual therapies can be applied to affect the visceral mobility and viscoelasticity, reaching repercussions on the activity of the tissue receptors and the sensitization state of the nervous system due to the therapeutic touch.

Visceral stimulation causes fluctuation in autonomic activity in brain areas involved in emotions [61]. In a study with volunteers diagnosed with irritable bowel syndrome, the osteopathic treatment focusing on the spine and abdominal viscera decreased the severity of the irritable bowel syndrome and also the psychological findings. The sham group, which received a light touch in the same places as the treated group, also showed improvement in the psychological variables evaluated [62].

In the study by Gürsen with volunteers diagnosed with intestinal constipation, there was an improvement in bowel function, pain and a decrease in psychosocial discomfort after manual treatment of the soft tissues of the pelvis and spine regions [63]. Some studies have demonstrated an improvement in flexibility and visceral function after manual treatment, which can impact on peripheral sensitization and interoception. Still's technique applied to the kidney generates increased renal mobility and decreased back pain in volunteers with low back pain [60]; mobilization of the sigmoid colon of asymptomatic volunteers generates hypoalgesia at the L1 level and the OMT, including visceral treatment, improves intestinal constipation, including stroke survivors [64-66].

The improvement in movement and visceral viscoelasticity probably interferes positively in spinal cord reflexes [67], and the mechanical tension due to the continuity of connective tissue [60], which would decrease dysfunctional interoceptive afferences. Therapeutic touch can positively influence peripheral sensitization via interaction with peripheral tissues [11]. Studies suggest that touch during OMT activates the insular cortex and generates interoceptive integration [68,69].

Touch is integrated, in the insular and cingulate cortex, with the information of interoception, exteroception, including pain, taste and smell, temperature, muscular work, thirst, dyspnoea, cardiorespiratory activation, vascular flush, and tension/distension of the oesophagus, stomach, bladder and rectum [69].

Like the soft touch, the static touch, performed slowly, widely used in visceral osteopathic treatment, generates depolarization of the C fibers, which transmit information to laminae 1 and 2 of the spinal cord, accessing the interoceptive pathway [3].

Edwards et al. found an increase in interoceptive accuracy after intervention in the group submitted to the deep touch over the cranial base and also in the group submitted to the mobilization of the temporomandibular joint [70]. Similar results were found by Cathcart after performing a myofascial release technique applied to the extensor muscles of the thoracic spine [71]. As the interoceptive pathway is the same to some extent, similar responses can be expected after visceral tissue interventions.

Pain inhibition can be another positive factor in these perspectives. The touch that accesses the C fibers generates pain inhibition [72], modifying sensitization states [11] and interfering in the mechanical hypersensitivity process [73]. This probably occurs because pain can be considered as an extension of interoceptive processing [74], promoting emotional, hormonal and behavioral responses [75].

It is worth noting that the interoceptive pathway can be accessed with different touch intensities because there is also top-down cognitive influences on affective representations of the individuals being touched [65].

## Conclusions

The manual treatment applied to the visceral tissues seems to be a possibility to contribute to the treatment of some emotional imbalances. The prospect to positively affect the function of visceral tissue receptors and consequently on the sensitization state of the nervous system could be plausibly related to a better integration of the interoception functions, which are closely related to some emotional behaviour. We suggest conducting experimental studies that analyze the responses of manual approaches to dysfunctional visceral tissues in conditions related to emotional behavior.
